# Lignin biosynthesis in wheat (*Triticum aestivum* L.): its response to waterlogging and association with hormonal levels

**DOI:** 10.1186/s12870-016-0717-4

**Published:** 2016-01-25

**Authors:** Tran-Nguyen Nguyen, SeungHyun Son, Mark C. Jordan, David B. Levin, Belay T. Ayele

**Affiliations:** Department of Plant Science, University of Manitoba, 222 Agriculture Building, Winnipeg, MB R3T 2N2 Canada; Morden Reasearch and Development Centre, Agriculture and Agri-Food Canada, Morden, MB R6M 1Y5 Canada; Department of Biosystems Engineering, University of Manitoba, Winnipeg, MB R3T 5V6 Canada

**Keywords:** Gene expression, Plant hormone, Lignin, Lodging, Waterlogging, Wheat

## Abstract

**Background:**

Lignin is an important structural component of plant cell wall that confers mechanical strength and tolerance against biotic and abiotic stressors; however it affects the use of biomass such as wheat straw for some industrial applications such as biofuel production. Genetic alteration of lignin quantity and quality has been considered as a viable option to overcome this problem. However, the molecular mechanisms underlying lignin formation in wheat biomass has not been studied. Combining molecular and biochemical approaches, the present study investigated the transcriptional regulation of lignin biosynthesis in two wheat cultivars with varying lodging characteristics and also in response to waterlogging. It also examined the association of lignin level in tissues with that of plant hormones implicated in the control of lignin biosynthesis.

**Results:**

Analysis of lignin biosynthesis in the two wheat cultivars revealed a close association of lodging resistance with internode lignin content and expression of *4-coumarate:CoA ligase1* (*4CL1*), *p-coumarate 3-hydroxylase1* (*C3H1*), *cinnamoyl-CoA reductase2* (*CCR2*), *ferulate 5-hydroxylase2* (*F5H2*) and *caffeic acid O-methyltransferase2* (*COMT2*), which are among the genes highly expressed in wheat tissues, implying the importance of these genes in mediating lignin deposition in wheat stem. Waterlogging of wheat plants reduced internode lignin content, and this effect is accompanied by transcriptional repression of three of the genes characterized as highly expressed in wheat internode including *phenylalanine ammonia-lyase6* (*PAL6*), *CCR2* and *F5H2*, and decreased activity of PAL. Expression of the other genes was, however, induced by waterlogging, suggesting their role in the synthesis of other phenylpropanoid-derived molecules with roles in stress responses. Moreover, difference in internode lignin content between cultivars or change in its level due to waterlogging is associated with the level of cytokinin.

**Conclusion:**

Lodging resistance, tolerance against biotic and abiotic stresses and feedstock quality of wheat biomass are closely associated with its lignin content. Therefore, the findings of this study provide important insights into the molecular mechanisms underlying lignin formation in wheat, an important step towards the development of molecular tools that can facilitate the breeding of wheat cultivars for optimized lignin content and enhanced feedstock quality without affecting other lignin-related agronomic benefits.

**Electronic supplementary material:**

The online version of this article (doi:10.1186/s12870-016-0717-4) contains supplementary material, which is available to authorized users.

## Background

Lignin is a complex phenolic polymer closely linked with cellulose and hemicellulose, forming an important structural component of plant secondary cell wall. It provides plants with mechanical strength and vascular integrity [1], and also plays important roles in conferring tolerance against biotic and abiotic stresses [2, 3]. Recently, increased concerns about climate change and the need to reduce carbon emissions have triggered a growing interest in producing renewable fuels and bioproducts from lignocellulosic biomass [4]. Wheat is one of the most economically important crops globally, and its straw represents an abundant source of biomass that can be used as a feedstock for sustainable production of biofuel and bioproducts [5, 6].

Efficient conversion of lignocellulosic biomass to biofuels is hindered by lignin, which limits the accessibility of plant cell wall polysaccharides to chemical, enzymatic and microbial digestions [7, 8]. Genetic alteration of the quantity and quality of lignin in plant biomass has been considered as a viable alternative to mitigate this problem [9–11]. However, lignin content in cereal crops such as wheat has been shown to be closely associated with resistance to lodging [12], one of the major impediments of wheat production leading to harvestable yield loss by up to 80 % [13]. Therefore, it is imperative to design tools and approaches that can alter the quantity and quality of lignin in wheat biomass without affecting its functions in conferring structural support for normal growth and development, and field performance. This, however, requires detailed dissection of the molecular mechanisms underlying the formation of lignin in wheat biomass.

**Fig. 1:**
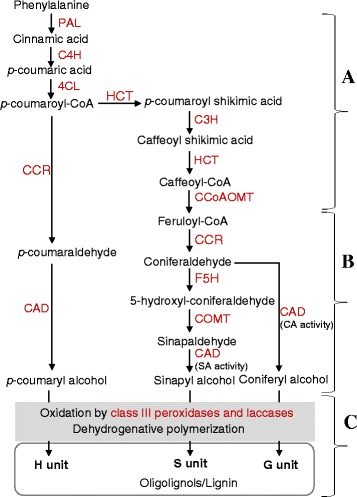
Lignin biosynthesis pathway in plants. The monolignols (coniferyl alcohol, sinapyl alcohol, and *p*-coumaryl alcohol) synthesized from phenylalanine through the general phenylpropanoid pathway (*A*) and monolignol-specific pathway (*B*) are oxidized and incorporated into the G (guaiacyl), S (syringyl) and H (hydroxyphenyl) units, respectively, in the complex and three-dimensional polymer of lignin (*C*). Oligolignols, which are formed during lignin polymerization, are racemic radical coupling products of monolignols. PAL, phenylalanine ammonia-lyase; C4H, cinnamate 4-hydroxylase; 4CL, 4-coumarate:CoA ligase; C3H, p-coumarate 3-hydroxylase; HCT, p-hydroxycinnamoyl-CoA:quinate/shikimate p-hydroxycinnamoyltransferase; CCoAOMT, caffeoyl-CoA O-methyltransferase; CCR, cinnamoyl-CoA reductase; F5H, ferulate 5-hydroxylase; COMT, caffeic acid O-methyltransferase; CAD, cinnamyl alcohol dehydrogenase

It has been established that lignin in plants is formed by the oxidative coupling of three monolignols that serve as building blocks; coniferyl, sinapyl and *p*-coumaryl alcohols [1]. These monolignols are synthesized from phenylalanine through the general phenylpropanoid and monolignol-specific pathways (Fig. 1), and the successive dehydrogenative polymerization reactions give rise to guaiacyl (G), syringyl (S) and hydroxyphenyl (H) units, respectively, that form a complex and three-dimensional lignin polymer [1]. In addition, lignin formation involves the incorporation of other monomers such as hydroxycinnamyl acetates, hydroxycinnamyl *p*-hydroxybenzoates, and hydroxycinnamyl *p*-coumarates under specific genetic and environmental conditions [14].

Lignin biosynthesis has been studied extensively in the model plant Arabidopsis and other dicot species such as alfalfa and tobacco, and these studies have led to the identification of genes encoding the enzymes catalyzing many of the steps in the pathway (Fig. 1) [1]. Since they catalyze the first committed step in the general phenylpropanoid pathway and the last step in the synthesis of monolignols, the PAL and CAD enzymes, respectively, are belived to play critical roles in regulating lignin accumulation in plants [15–17]. Given that most of the lignin biosynthetic enzymes have isoforms, they appear to be encoded by gene families [18, 19], and previous studies have demonstrated the physiological roles for most of these genes in determining lignin deposition and composition [1, 10]. For example, suppression of genes early in the pathway such as *PAL*, *C4H*, *HCT*, *C3H* and *CCoAOMT* in alfalfa and tobacco leads to reduction in total lignin content, while repression of *F5H* or *COMT*, which are involved in the synthesis of the S unit of lignin, results in alteration of the S/G ratio of lignin with only minimal effect on total lignin content [9]. Genetic repression of *CAD*, encoding an enzyme that catalyzes the last step in the biosynthesis of monolignols, also causes reduction in the S/G ratio with limited effect on total lignin content. Similarly, the *f5h1* and *comt* mutants of Arabidopsis are characterized by decreased amounts of S unit containing oligolignols with no effect on the total lignin content, whereas the *c4h*, *4cl1*, *ccoaomt1*, and *ccr1* mutants contain lower amount of total lignin as compared to the wild-type [20].

Although lignin biosynthesis pathway has not been elucidated in wheat to date, previous studies have characterized the expression patterns of the wheat homologs of lignin biosynthetic genes. The transcripts for most of these homologs appeared to be highly abundant in the stem tissue as compared to the leaf sheath and leaf blade, and the expressions of *PAL6*, *C4H*, *4CL1*, *C3H1*, *CCR2*, *F5H1* and *F5H2* in particular are found to have a significant correlation with lignin content [3]. Furthermore, the stem exhibits higher abundance of *CCR1* and *CAD1* transcripts and higher activity of the corresponding enzymes than that detected in other tissues, while *COMT1* is expressed constitutively across the different tissues including stem, leaf and root [12, 21, 22]. Comparative analysis between wheat cultivars with varying degree of resistance to lodging showed the presence of higher transcript abundance of *CCR1*, *COMT1* and *CAD1* genes and higher activities of the corresponding enzymes in the stem of lodging resistant cultivar following the heading stage, and these factors have been shown to be closely associated with stem lignin content and mechanical strength [21]. Despite these results, the molecular mechanism underlying the synthesis of lignin in wheat tissues is yet poorly understood.

Waterlogging is one of the abiotic factors adversely affecting crop productivity; it causes a reduction in gas diffusion and thereby affects the availability of oxygen in the rhizosphere, inducing changes in biochemical and metabolic processes [23]. One of the main metabolic changes involves a shift from aerobic to anaerobic respiration, impairing ATP production. Compensation of the resulting energy deficit requires accelerated glycolysis via increased activities of glycolytic and fermentative enzymes, leading to the depletion of carbohydrate reserves, a phenomenon referred to as the “Pasteur effect” [24, 25]. Consistently, transcriptional activation of glycolytic and fermentative genes has been reported in rice coleoptiles under anoxic conditions [26]. In contrast with this, root hypoxic conditions have been shown to result in accumulation of soluble carbohydrates such as sucrose, and those with storage function such as fructan in both root and shoot tissues of wheat seedlings [27]. Under long-term hypoxic conditions, a large amount of sucrose in wheat roots partitions to the synthesis of cell wall components, mainly cellulose, leading to changes in cell wall structure [28]. It is therefore likely that such alteration in the structure of the cell wall serves as one of the strategies used by the root tissue to compensate the progressive dissolution of cortical cells for aerenchyma formation, thereby contributing to the maintenance of cell wall function under low O_2_ stress conditions [29]. However, little is known to date about the molecular features mediating the response of lignin biosynthesis to waterlogging conditions.

In addition to environmental factors, cellular signaling molecules such as plant hormones regulate lignin biosynthesis [30, 31]. For example, auxin and cytokinin induce the expression of the lignin biosynthetic gene, *peroxidase* (*Prx*), in *Zinnia elegans*, and secondary growth/lignification [30]. Consistently, the promoter of *Prx* consists of motifs that mediate the response of *Prx* to these hormones and thereby act as targets for transcription factors regulating secondary growth [30, 32]. Furthermore, hypergravity-induced auxin accumulation leads to enhanced expression of selected lignin biosynthetic genes, and in turn lignification in the inflorescence stem of Arabidopsis [33]. It has been shown previously that the level of salicylic acid (SA) is inversely related to lignin content in plants where lignin content is reduced through downregulation of specific lignin biosynthetic genes, and SA mediates growth supression in these plants; however, genetic reduction of SA level was found to restore growth but not lignin content [34].

To gain insights into the molecular mechanisms underlying the regulation of lignin formation in wheat, we performed comparative analysis of lignin biosynthesis in the internode tissue of wheat cultivars with varying degree of resistance to lodging, a trait closely associated with stem lignin content and mechanical strength. Furthermore, the study identified candidate genes that mediate the response of lignin biosynthesis in wheat tissues to waterlogging, and examined the association between the levels of lignin and selected plant hormones that have been implicated to have roles in the regulation of lignin biosynthesis.

## Methods

### Plant material

Two hexaploid wheat cultivars, Harvest and Kane, were used in this study, and seeds for these cultivars were kindly provided by Dr. Stephen Fox of Agriculture and Agri-Food Canada (AAFC)-Cereal Research Center (Winnipeg, Manitoba, Canada). Both are hard red spring wheat cultivars developed by AAFC [35, 36], and were selected for the present study on the basis of their agronomic quality in terms of lodging resistance, which is considered as a measure of stem mechanical strength, and their close genetic relationship. Mature dry seeds of the two cultivars were imbibed in Petri dishes for 3 days, after which the germinated seeds were transplanted to 1-gallon plastic pots (one seed per pot) containing Super Mix supplied with 18 g of fertilizers (ACER®nt 13-12-12 consisting of 13 % N, 12 % P_2_O_5_, 12 % K_2_O and micro elements). Plants were grown in a growth chamber at 22/20 °C (day/night) under a 16/8 h photoperiod until harvest. At the heading stage, two sets of samples were collected: the first set was harvested from cv. Harvest and consisted of flag leaf blade, flag leaf sheath, peduncle, first internode (IN-1), a combination of second and third internodes (IN-2&3) and the fourth internode (IN-4). Numerical designation of the internodes was based on their relative position to the peduncle in which IN-1 is the youngest internode located next to the peduncle, while IN-4 is the oldest internode located next to the base of the stem. The second set of samples was harvested from the two cultivars, cvs. Harvest and Kane, and consisted of the whole flag leaf (both leaf blade and sheath) and the internode (IN-2&3). Tissues were frozen in liquid nitrogen immediately after harvest and then stored at −80 °C until further use.

### Waterlogging treatment

For waterlogging treatment, plants of cv. Harvest were grown as described above except that the soil mixture consisted of clay and sand (2:1, v:v). Four weeks after transplanting, a set of 48 plants was subjected to waterlogging by submerging each pot containing a plant in a 5 L containers filled with water in order to maintain the water level at 2 cm above the surface of the soil. The water in the bigger container was replenished as required. Concurrently, another set of 48 plants was subjected to regular watering (0.5 L water per pot every other day). At heading stage, which occurred at 20 to 22 days after the start of waterlogging in both treated and untreated control plants, the flag leaf and IN-2&3 tissues were harvested in liquid nitrogen and stored at −80 °C until further use.

### Identification of candidate lignin biosynthetic genes and phylogenetic analysis

The tentative consensus (TC) sequences of candidate lignin biosynthetic genes from the Dana-Farber Cancer Institute (DFCI) wheat gene index (TaGI) release 10 and 11 [3] were used to search for the corresponding TCs in TaGI release 12 (ftp://occams.dfci.harvard.edu/pub/bio/tgi/data/Triticum_aestivum/) [37]. The GenBank IDs corresponding to the newly identified TCs were used to search the National Center for Biotechnology Information (NCBI) wheat UniGene dataset containing 56,943 unigenes (http://www.ncbi.nlm.nih.gov/UniGene/UGOrg.cgi?TAXID=4565) [38] to obtain the respective unigenes, which consist of a cluster of sequences representing a unique lignin biosynthetic gene family member. A complete coding sequence (CDS) from each sequence cluster or a TC sequence from release 12 (when a complete CDS is not available) was used as a query to blast search the NCBI GenBank nucleotide database (http://www.ncbi.nlm.nih.gov/nuccore/) [39] for additional family members from wheat (derived from a different unigene) or for homologous genes from other species using the criteria of at least 50 % coverage of the query sequence and E-value of ≤10^−20^. Sequences meeting these criteria were collected for each lignin biosynthetic gene family and used to generate phylogenetic trees. The sequences were collected mainly from rice (*Ozyza sativa*, taxid 4530), sorghum (*Sorghum bicolor*, taxid 4558), barley (*Hordeum vulgare*, taxid 4513), switchgrass (*Panicum virgatum*, taxid 38727), maize (*Zea mays*, taxid 4577), and other species for which candidate lignin biosynthetic gene sequence information is available. Sequences of the Arabidopsis lignin biosynthetic genes were collected based on the protein sequences reported in Xu et al. [19]. The nucleotide sequences of the genes were then aligned using ClustalW program (http://www.ebi.ac.uk/Tools/msa/clustalw2) [40] and the phylogenetic tree was generated with the Molecular Evolutionary Genetic Analysis (MEGA, version 6) software (http://www.megasoftware.net) [41] using a Tamura-Nei model and a 500 replicate bootstrap method of phylogeny test.

### RNA extraction and cDNA synthesis

Total RNA was isolated from tissues using TRIzol Reagent following the manufacturer’s protocol (Life Technologies, Carlsbad, CA, USA). The RNA samples were digested with DNase (DNA-free Kit; Ambion, Austin, TX, USA) to eliminate genomic DNA contamination. The first strand cDNA was synthesized from 1 μg of total RNA using iScript Reverse Transcription Supermix (Bio-Rad, Hercules, CA, USA) in a total reaction volume of 20 μl. The resulting cDNA samples were diluted 20× before use as a template for real-time quantitative PCR (qPCR).

### Real-time quantitative PCR assay

The real time qPCR assays for the candidate lignin biosynthetic and the reference *β*-*actin* genes were performed with the gene specific primers described previously [3] except for the new candidate genes identified in this study and the genes for which redesigning the primers was required due to their lack of target gene specificity (Additional file 1: Table S1). Gene specificity of all the primers was verified first by blast searching the target amplicons against GenBank database and then by RT-PCR. The qPCR assay was performed on the CFX96 Real-Time PCR system (Bio-Rad) and the reaction consists of 5 μL of the diluted cDNA as a template, 10 μL of SsoFast EvaGreen Supermix (Bio-Rad), 1.2 μL of 5 μM forward primer (300 nM final concentration), 1.2 μL of 5 μM reverse primer (300 nM final concentration) and 2.6 μL diethylpyrocarbonate treated water. The samples were subjected to the following thermal cycling conditions: DNA polymerase activation at 95 °C for 5 min followed by 40 cycles of denaturation at 95 °C for 15 s, annealing at 50–66 °C (depending on the melting temperature of the primer set) for 30 s and extension at 72 °C for 30 s in duplicate in 96-well optical reaction plates (Bio-Rad). Transcript levels of the target genes were expressed after normalization with *β*-*actin* using the Livak and Schmittgen method [42].

### Measurement of enzyme activity

Protein concentrations and enzyme activities were determined spectrophotometrically (Ultrospec 3100 *pro*, Artisan Scientific, Champaign, IL, USA). Total protein extraction, and the assays for determining the activities of coniferyl aldehyde recognizing CAD (CAD-CA) and sinapyl aldehyde recognizing CAD (CAD-SA) were carried out as described in Zhang et al. [43]. Briefly, frozen plant tissue (~200 mg of flag leaf or ~300 mg of internode) was ground into fine powder using mortar and pestle, mixed with protein extraction buffer and then incubated at 4 °C for 2.5 h. Protein concentration in the extract was measured using Quick Start™ Bradford Protein Assay (Bio-Rad, Hercules, CA, USA). The CAD-CA and CAD-SA activities were examined by using total protein extract (100 μg) in 500 μL reaction buffer and monitoring the conversion of coniferyl alcohol, a substrate for CAD-CA, and sinapyl alcohol, a substrate for CAD-SA, to their respective products at 400 nm. The activity of PAL in the protein extract was assayed as described in Edwards and Kessmann [44] in which the formation of cinnamic acid from L-phenylalanine was monitored at 290 nm for up to 2 h using total protein extract (100 μg for flag leaf or 10 μg for internode) in 1 mL reaction buffer. The activity for each enzyme was expressed as nKatal (referring to the enzyme activity required for converting 1 nmol of the substrate per second) per gram of total protein.

### Measurement of lignocellulosic constituents

Frozen leaf and internode tissue samples were lyophilized, and then subjected to analysis of the lignocellulosic constituents, which was performed at the Feeds Innovation Institute of the University of Saskatchewan (Saskatoon, Saskatchewan, Canada). Lignin and acid detergent fiber (ADF) contents were profiled using the Association of Official Analytical Chemists (AOAC) Method 973.18 [45] while the neutral detergent fiber (NDF) content was determined using the AOAC Method 2002.04 [45]. Cellulose content was expressed as the difference between ADF and lignin contents; while the hemicellulose content was expressed as the difference between NDF and ADF contents.

### Measurement of hormone levels

Hormone levels were measured from the same tissue samples used for analysis of gene expression and the levels of lignocellulosic constituents. Auxin (indole acetic acid [IAA]), cytokinin (isopentenyl adenine [IPA] and trans-zeatin [t-zeatin]) and salicylic acid (SA) were extracted from the internode tissues as described previously [46] except that the intial extraction was performed with 80 % (v/v) acetonitrile containing 1 % (v/v) acetic acid and elutions of the IAA and SA extracts from the respective columns were performed with 80 % (v/v) acetonitrile containing 1 % (v/v) acetic acid while the IPA and t-zeatin extracts were eluted with 60 % (v/v) acetonitrile containing 5 % (v/v) aqueous ammonia. Quantification of the IAA, IPA and t-zeatin, and SA levels was performed using liquid chromatography-tandem mass spectrometery system (Agilent 6430) (Agilent, Santa Clara, CA, USA) according to the protocol described in Yoshimoto et al. [47].

### Statistical analysis

Significant difference between sample means was tested using either LSD test or t-student test at *P* < 0.05. Statistical analysis was performed with GenStat version 12 [48].

## Results

### Update on the wheat homologs of lignin biosynthetic genes

**Fig. 2:**
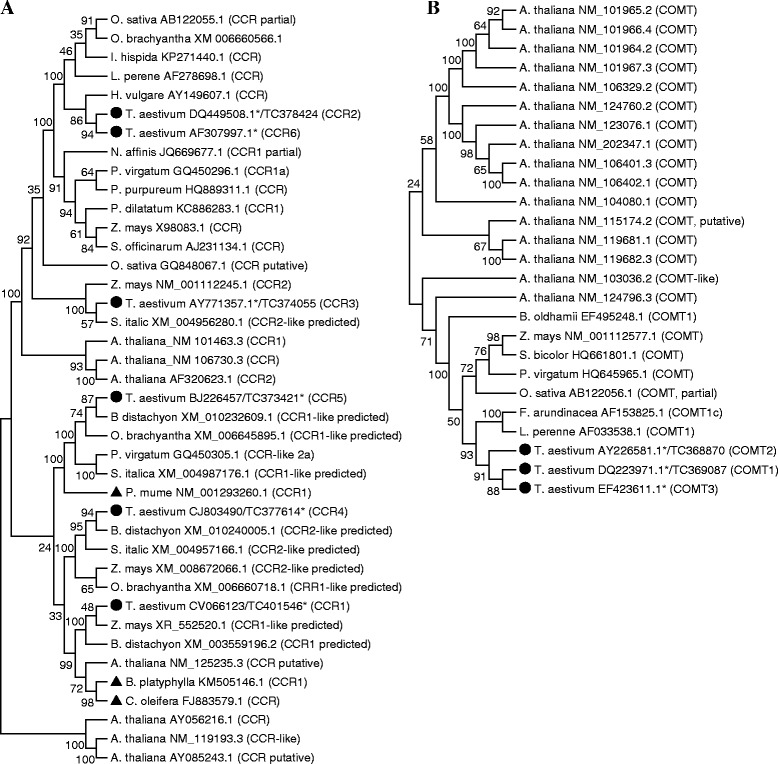
Phylogenetic relationships of wheat *CCR* and *COMT* genes with the homologs from other species. Phylogenetic trees of *CCR* (**a**) and *COMT* (**b**) were generated based on nucleic acid sequence similarity of the wheat genes with 34 *CCR* and 23 *COMT* genes, respectively, of other monocot and dicot species identified from the NCBI nucleotide database [39] using MEGA program [41], and the trees were inferred using Maximum Likelihood method based on the Tamura-nei model. The percentage of replicate trees in which the associated taxa clustered together in the bootstrap test of 500 replicates is shown next to the branches. ●, wheat candidate gene; ▲, genes from dicot species other than Arabidopsis; *, wheat sequence used for the analysis

Using the TaGI release 10 and 11, Bi et al. [3] identified 32 candidate lignin biosynthetic genes belonging to 10 gene families; however only two of them, *COMT1* and *COMT2*, were found to have sequences that represent complete CDS in the respective cluster of sequences. Since the GenBank database has been updated with more wheat gene sequences and the TaGI release 12 consisting of 221,925 TCs has been made available since the report of Bi et al. [3], we performed further database mining and updated the list of the candidate wheat lignin biosynthetic gene (Table 1). Our search revealed that specific ESTs assigned by Bi et al. [3] to different gene family members appear to originate from the same unigene, thus represent the same gene family member instead of different members. ESTs designated in Bi et al. [3] as *4CL1* and *4CL2* are found to belong to the same unigene (Ta. 45532), thus represent the same *4CL* gene designated hereafter as *4CL1*; ESTs designated as *CCoAOMT1* and *CCoAOMT4* come from the same unigene (Ta. 18653), thus represent the same *CCoAOMT* gene designated hereafter as *CCoAOMT1*; ESTs designated as *CCoAOMT3* and *CCoAOMT5* originate from the same unigene (Ta. 48354), thus represent the same *CCoAOMT* gene designated hereafter as *CCoAOMT3*, and ESTs designated as *PAL2*, *PAL3* and *PAL4* derive from the same unigene (Ta. 47240), thus represent the same *PAL* gene designated hereafter as *PAL2*. Accordingly, the ESTs identified by Bi et al. [3] as *PAL5*, *6*, *7* and *8* are redesignated as *PAL3*, *4*, *5* and *6*, respectively. The selection of a particular EST for the designation in each family member was based on their coding region coverage, and these specific ESTs were considered for our gene expression analysis (Table 1). Furthermore, the EST assigned by the same authors as *CAD2* is found to be a homolog of the *CCR* gene, thus it is re-designated hereafter as *CCR5* (Table 1 and Fig. 2a). Our search also led to the identification of new candidate lignin biosynthetic genes belonging to the *C3H* (designated as *C3H3*), *CCR* (designated as *CCR6*) and *COMT* (designated as *COMT3*) gene families (Table 1).

**Table 1:** Updated list of the candidate lignin biosynthesis gene family members of wheat

Name	TC ID^a^	UniGene ID^b^	GenBank ID^c^
*PAL1*	TC383948	Ta.70840	CJ956144
*PAL2*	TC414011	Ta.47240	CJ873958
*PAL3*	TC385013	Ta.70842	CJ963731
*PAL4*	TC369518	Ta.48480	CJ954700
*PAL5*	TC405175	Ta.71604	CJ964755
*PAL6*	TC385356	Ta.21253	CJ728010
*C4H1*	TC384685	Ta.253	CK157495
*4CL1*	TC395589	Ta.45532	CJ962785
*HCT1*	TC396512	Ta.37502	CK193498
*HCT2*	TC396512	Ta.5332	CK199765
*C3H1*	TC372953	Ta.24789	**AJ583530.1**, **AJ583531.1** ^d^
*C3H2*	TC368628	Ta.31019	**AJ585988.1**, **AJ585990.1**, **AJ585991.1**
*C3H3*	NA^e^	Ta.48868	**AJ583532.1**
*CCoAOMT1*	TC374467	Ta.18653	CD939543
*CCoAOMT2*	TC373325	Ta.39255	CJ928722
*CCoAOMT3*	TC398408	Ta.48354	CJ966710
*CCR1*	TC401546	Ta.12690	CV066123
*CCR2*	TC378424	Ta.1183	**DQ449508.1**
*CCR3*	TC374055	Ta.13990	**AY771357.1**
*CCR4*	TC377614	Ta.71133	CJ803490
*CCR5*	TC373421	Ta.48506	BJ226457
*CCR6*	NA	Ta.58638	AF307997
*F5H1*	TC410836	Ta.73421	CA684885
*F5H2*	TC383087	Ta.70215	CJ962608
*COMT1*	TC369087	Ta.70123	**DQ223971.1**, **EF413031.1**
*COMT2*	TC368870	Ta.336	**AY226581.1**
*COMT3*	NA	Ta.41808	**EF423611.1**
*CAD1*	TC445835	Ta.32577	CJ710661
*CAD2*	TC374092	Ta.70656	CJ723808
*CAD3*	TC379496	Ta.28562	**GU563724.1**

^a^TC ID, tentative consensus sequences ID (DFCI TaGI 12.0) [37]

^b^UniGene ID, NCBI-wheat UniGene ID [38]

^c^GenBank ID, NCBI GenBank ID [39]

^d^GenBank IDs in bold represent complete coding sequences

^e^ *NA* not available

### Phylogenetic analysis of the candidate lignin biosynthesis genes

Phylogenetic analysis of the wheat candidate lignin biosynthetic genes along with the representative homologs from other species (9 to 34 genes depending on the gene family) revealed that each wheat candidate gene is clustered with more than one homolog derived from the other species except that all the three wheat *COMT* genes formed their own group (Fig. 2; Additional file 2: Figure S1, Additional file 3: Figure S2, Additional file 4: Figure S3 and Additional file 5: Figure S4). In contrast, the six *CCR* genes were distributed among five different clades (Fig. 2b). Overall, most of the wheat lignin biosynthetic genes are grouped mainly with their homologs that originate from monocot species instead of those derived from Arabidopsis and other dicot species (Fig. 2; Additional file 2: Figure S1, Additional file 3: Figure S2, Additional file 4: Figure S3 and Additional file 5: Figure S4).

### Lodging behavior of the wheat cultivars

Harvest and Kane are among the cultivars widely grown in the wheat growing regions of the Canadian Prairies [49]. These two cultivars are closely related genetically as they are developed by a cross that involved a common parental line; Kane is derived from a cross between AC Domain and McKenzie [35], and Harvest from a cross between AC Domain*2 and ND640 [36] (*2 denotes two doses of AC Domain; one backcross after the original cross). With respect to their resistance to lodging, Kane and Harvest are rated as ‘very good’ and ‘good’, respectively [50, 51]. It can also be inferred from the registration data of Harvest and Kane, which compared each cultivar against the same check cultivar over three years at multiple locations, that Harvest exhibits lower lodging score than Kane; lodging rated on a 1–9 scale, 1 = vertical and 9 = flat [35, 36]. Kane exhibited consistently similar lodging scores to the check cultivars AC Barrie (avergae lodging score of 1.7 in year I, 2.1 in year II and 2.5 in year III for Kane, and 1.9 in year I, 2.2 in year II and 2.5 in year III for AC Barrie) [35]. In contrast, Harvest exhibited lower lodging score than the check cultivar AC Barrie (on avergae 2.0 in year I, 1.4 in year II and 1.7 in year III for Harvest, and 2.6 in year I, 2.4 in year II and 2.5 in year III for AC Barrie) [36]. As the studies that compared each cultivar against the common check cutivar were conducted at different times, the results also suggest that the severity of lodging was worse in years when the performance of Harvest was tested.

### Expression of the candidate lignin biosynthetic genes in different wheat tissues

The expression patterns of candidate lignin biosynthetic genes in different tissues and stages of wheat have been characterized previously [3]. Given that the list of the candidate genes has been updated with the latest databases (Table 1) and our experimental materials involve wheat cultivars different from that used by Bi et al. [3], we decided to re-examine the spatiotemporal expression pattern of all the candidate genes. Our analysis revealed that all the candidate genes showed higher expression in the peduncle and/or internode tissues than in the flag leaf blade/sheath except that *CCR1* and *CCR5* genes exhibited significantly higher expression in the flag leaf blade than in the other tissues analyzed in this study, and *COMT2* and *CAD2* in their respective gene family are found to be highly expressed in both flag leaf sheath and internode tissues (Additional file 6: Table S2). The *C3H2*, *CCoAOMT2* and *CCR4* genes are found to be expressed at very low/undetectable level across all the tissue analyzed.

**Fig. 3:**
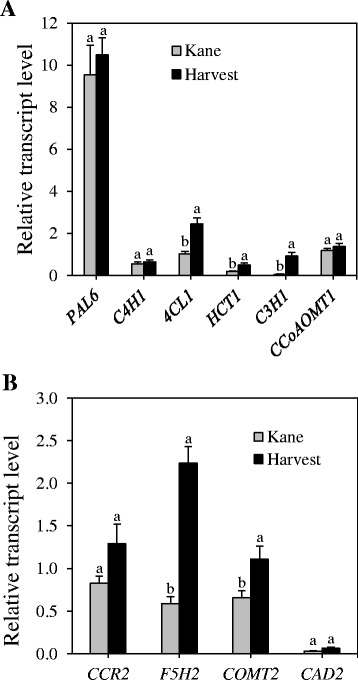
Expression of lignin biosynthesis genes highly expressed in wheat internode tissue. Relative transcript levels of internode-derived genes involved in the general phenylpropanoid (**a**) and monolignol specific (**b**) pathways were compared between the wheat cultivars Kane and Harvest; the internodes were collected at the heading stage. Transcript levels were expressed relative to that of *4CL1* in cv. Kane, which was arbitrarily set to a value of 1. Data are means of 2 to 3 independent biological replicates ± SE. Different *letters* within each gene show statistically significant difference in transcript level at *P* < 0.05

Specific members in each gene family including *PAL6*, *C4H1*, *4CL1*, *HCT1*, *C3H1*, *CCoAOMT1*, *CCR2*, *F5H2* and *COMT2* appeared to be highly expressed in the peduncle/internode (Table 2). From the *CAD* gene family, the transcripts of both *CAD1* and *CAD2* are highly abundant in the internode, although the transcript level of *CAD2* is slightly higher than that of *CAD1*. Comparative analysis across the different internode sections revealed that most of these genes are highly expressed in the IN-2&3 and/or IN-4 sections (Table 2). As a result, their expression in IN-2&3 was further compared between the two cultivars with varying degree of resistance to lodging, a trait closely associated with stem lignin content. It appeared from our analysis that *4CL1*, *HCT1*, *C3H1*, *F5H2* and *COMT2* genes exhibit significantly higher transcript level in the internode of cv. Harvest than in cv. Kane (Fig. 3). Although not statistically significant, higher expression of *CCR2* was also evident in the internode of cv. Harvest. The other four genes, *PAL6*, *C4H1*, *CCoAOMT1* and *CAD2*, exhibited similar expression level between the two cultivars.

**Table 2:** Relative transcript levels of lignin biosynthesis genes highly expressed in different tissues of wheat^w^

Gene	FB	FS	PE	IN-1	IN-2&3	IN-4
*PAL6*	0.83 ± 0.07^x^d^y^	9.05 ± 0.80c	89.25 ± 19.53a	54.50 ± 6.71b	54.57 ± 11.98b	33.66 ± 6.98b
*C4H1*	0.38 ± 0.03d	0.91 ± 0.04c	2.68 ± 0.40b	3.67 ± 0.61b	9.57 ± 1.20a	11.22 ± 3.71a
*4CL1*	1.00 ± 0.09d	2.04 ± 0.08c	7.61 ± 1.10b	7.13 ± 0.57b	12.51 ± 2.02a	16.84 ± 2.83a
*HCT1*	0.58 ± 0.03d	0.70 ± 0.06d	2.53 ± 0.54b	1.54 ± 0.20c	3.09 ± 0.40b	5.42 ± 0.60a
*C3H1*	0.12 ± 0.03e	0.21 ± 0.03de	1.24 ± 0.06a	0.87 ± 0.16b	0.55 ± 0.12c	0.46 ± 0.03 cd
*CCoAOMT1*	1.52 ± 0.17e	3.32 ± 0.58d	32.73 ± 5.28a	11.98 ± 2.51c	19.56 ± 3.51bc	21.89 ± 4.77b
*CCR1* ^*z*^	11.11 ± 0.21a	6.64 ± 0.36b	0.29 ± 0.03f	1.59 ± 0.31c	0.78 ± 0.02d	0.45 ± 0.07e
*CCR2*	0.17 ± 0.02e	1.56 ± 0.07d	3.77 ± 0.78c	5.93 ± 0.29b	8.44 ± 0.85a	7.82 ± 1.09a
*CCR5* ^*z*^	13.49 ± 1.10a	9.33 ± 0.21b	0.93 ± 0.09d	2.76 ± 0.66 cd	2.85 ± 0.36 cd	3.54 ± 0.63c
*F5H2*	0.84 ± 0.04e	1.39 ± 0.04d	9.20 ± 1.67a	6.09 ± 0.97ab	3.38 ± 1.05c	4.23 ± 0.84bc
*COMT2*	15.51 ± 1.13b	36.04 ± 4.20a	16.09 ± 2.86b	31.56 ± 4.40a	14.18 ± 1.64b	12.63 ± 0.67b
*CAD2*	1.32 ± 0.07b	2.05 ± 0.08a	0.16 ± 0.00c	0.95 ± 0.22b	2.02 ± 0.17a	2.55 ± 0.29a

^w^Transcript levels in different tissues of cv. Harvest were expressed relative to that of *4CL1* in flag leaf, which was arbitrarily set to value of 1

^x^Data are means of 2 to 3 independent biological replicates ± SE

^y^Means followed by different letters within each gene show statistically significant difference at *P* < 0.05

^z^Genes highly expressed specifically in the leaf tissues

*FB* flag leaf blade, *FS* flag leaf sheath, *PE* peduncle, *IN-1* the first internode, *IN-2&3* the second and third internode, *IN-4* the fourth internode (see the Methods section for numerical designation of internodes)

### Transcriptional response of candidate lignin biosynthetic genes to waterlogging

**Fig. 4:**
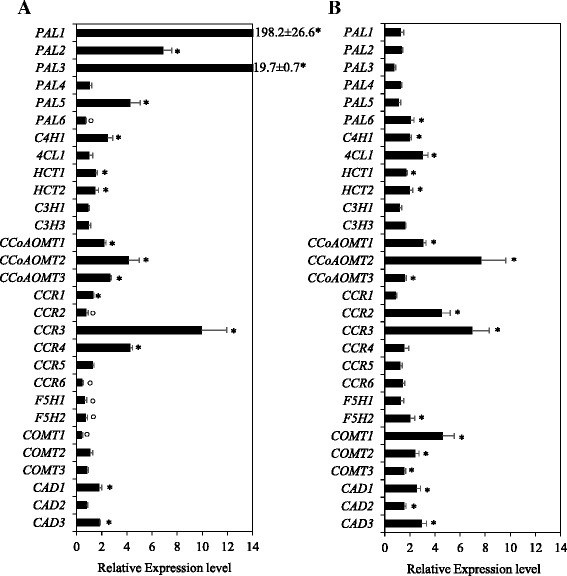
Expression of lignin biosynthesis genes in the internode and flag leaf in response to waterlogging. Relative transcript levels of each gene in the internode (**a**) and flag leaf (**b**) tissues of waterlogged plants of cv. Harvest were expressed relative to that detected in the corresponding tissue of the control plants, which was arbitrarily set to a value of 1. Data are means of 3 to 4 independent biological replicates ± SE. The * and ^○^ symbols indicate statistically significant upregulation and downregulation of the taget genes in response to waterlogging, respectively, as compared to that of the control at *P* < 0.05. *C3H2* was not analyzed as no transcript of this gene was detected in the internode (IN-2&3) and flag leaf tissues of wheat (see Additional file 6: Table S2)

In order to gain insights into the transcriptional regulation of lignin biosynthesis genes by waterlogging, we analyzed the expression of all the candidate genes in the internodes of waterlogged plants. Waterlogging led to a significant transcriptional repression of *PAL6*, *4CL2*, *CCR2*, *CCR6*, *F5H1*, *F5H2* and *COMT1* genes (Fig. 4a). On the other hand, all the *PAL* genes, except *PAL4* and *PAL6*, were upregulated in response to waterlogging; the expression of *PAL1* in particular was induced by ~200-fold. However, the basal expression level of this gene in the internodes of untreated control plants was relatively low (Additional file 6: Table S2). Other genes with ≥2-fold upregulation in response to waterlogging include *C4H1*, *CCoAOMT1*, *CCoAOMT2*, *CCoAOMT3*, *CCR3* and *CCR4*. The waterlogging treatment also induced upregulation (~1.8-fold) of *CAD1* and *CAD3* genes, although not ≥2-fold. However, the expressions of *PAL4*, *4CL1*, *C3H1*, *C3H3*, *COMT2*, *CCR5* and *CAD2* remained unaffected by the waterlogging treatment.

To better understand the effects of waterlogging on lignin synthesis, we also analyzed the expression of lignin biosynthesis genes in the flag leaf (blade plus sheath) of waterlogged plants. Waterlogging led to ≥2-fold upregulation of *PAL6*, *C4H1*, *4CL1*, *CCoAOMT1*, *CCoAOMT2*, *CCR2*, *CCR3*, *F5H2*, *COMT1*, *COMT2*, *CAD1* and *CAD3* genes (Fig. 4b), of which *PAL6*, *C4H1*, *4CL1*, *CCoAOMT1*, *F5H2* and *COMT2* are found to be the predominant genes in the leaf tissue in their respective family (Table 2; Additional file 6: Table S2). Although *CCoAOMT2* and *CCR3* showed drastic induction (over 7-fold) in response to waterlogging, their basal expression in the flag leaf of the control untreated plants was very low (Additional file 6: Table S2).

Waterlogging induced ≥2-fold upregulation of *PAL1*, *PAL2*, *PAL3*, *PAL5* and *CCR4* was evident in the internode but not in the flag leaf, where their expression appeared not to be affected by waterlogging (Fig. 4). In contrast, waterlogging led to ≥2-fold upregulation of *PAL6*, *4CL1*, *CCR2*, *F5H2*, *COMT1* and *COMT2* in the flag leaf but not in the internode, where their expression was either repressed or remained unaffected.

### Analysis of the activity of lignin biosynthesis enzymes

**Fig. 5:**
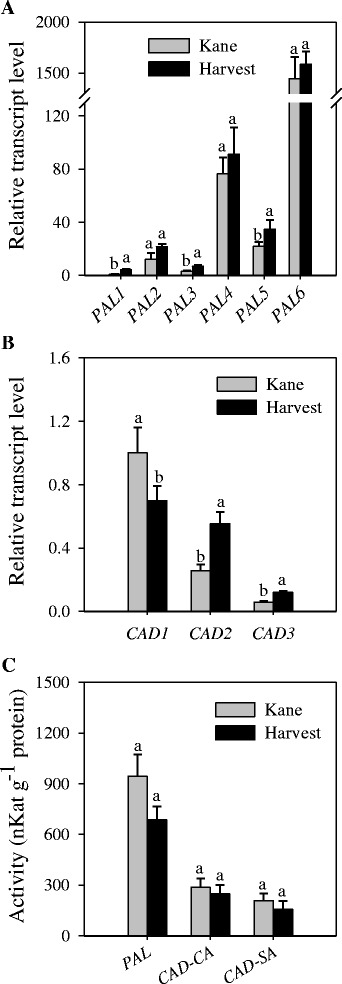
Expression of *PAL* and *CAD* genes and activity of the corresponding enzymes. Relative transcript levels of internode-derived *PAL* (**a**) and *CAD* (**b**) genes and activities of PAL, CAD-CA and CAD-SA enzymes (**c**) were compared between the wheat cultivars Kane and Harvest; the internodes were collected at the heading stage. Transcript levels were expressed relative to that of *PAL1* (**a**) and *CAD1* (**b**) in cv. Kane, which were arbitrarily set to a value of 1. Data are means of 2 to 3 independent biological replicates ± SE. Different *letters* within each gene and enzyme show statistically significant difference in activity at *P* < 0.05

**Fig. 6:**
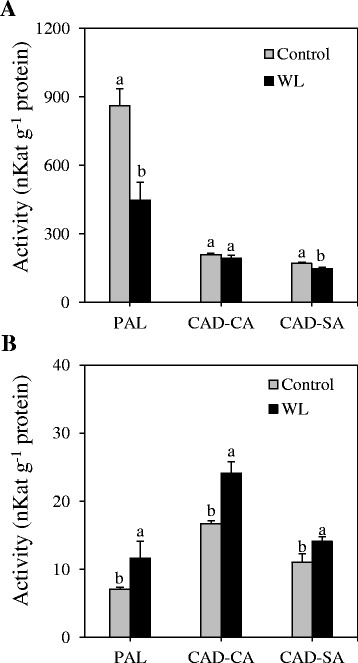
Changes in the activities of wheat lignin biosynthetic enzymes in response to waterlogging. The activities of PAL, CAD-CA and CAD-SA enzymes were measured in the internodes (**a**) and flag leaf (**b**) tissues of wheat cv. Harvest plants grown under waterlogging (WL) and control conditions. Data are means of 2 to 3 independent biological replicates ± SE. Different *letters* within each enzyme show statistically significant difference in activity at *P* < 0.05

To gain insights into the association between transcriptional and post-transcriptional regulations of genes encoding key lignin biosynthetic enzymes, we measured the activities of PAL catalyzing the first committed step in the phenylpropanoid pathway and CAD catalyzing the last step in the monolignol pathway (Fig. 1). Our data showed slightly higher activity of internode-derived PAL in cv. Kane than in cv. Harvest (Fig. 5c). The activity of CAD-SA and CAD-CA in the internode was found to be similar between the two cultivars. Waterlogging led to significant reduction in the activities of internode-derived PAL and CAD-SA enzymes although no effect was evident on the activity of CAD-CA (Fig. 6a). However, it caused significant increases in the activities of flag leaf-derived PAL, CAD-CA, and CAD-SA enzymes (Fig. 6b).

### Analysis of the major lignocellulosic constituents

**Fig. 7:**
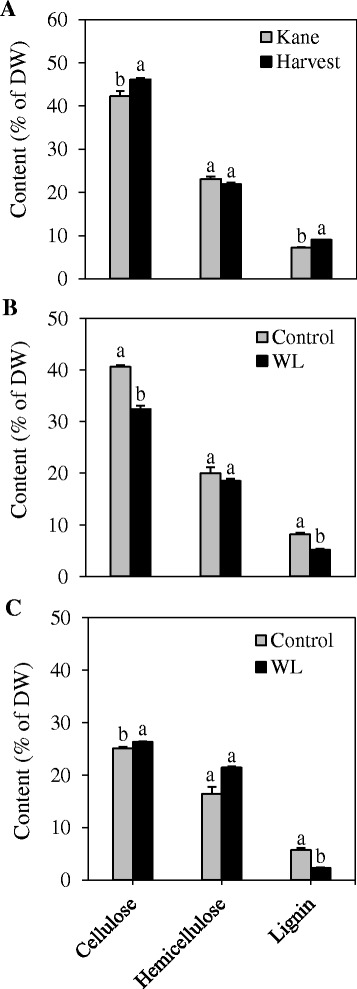
Profiles of the major constituents of lignocellulose. Cellulose, hemicellulose and lignin levels were determined in the internodes of the two wheat cultivars Kane and Harvest (**a**), and in the internode (**b**) and leaf (**c**) tissues of cv. Harvest in response to waterlogging (WL). Data are means of 2 to 3 independent biological replicates ± SE. Different *letters* within each lignocellulosic constituent show statistically significant difference in level at *P* < 0.05. Plant materials to study differences in the amounts of the major constituents of lignocellulose between the two cultivars were grown in Super Mix (see Methods) while plant materials to study the effect of waterlogging on the level of the major constituents of lignocellulose were grown in soil mixture of clay and sand (2:1, v:v)

To determine the association of stem resistance to lodging with the level of lignin and other cell wall components including cellulose and hemicellulose, we profiled their levels in the internode tissue of cvs. Harvest and Kane. Internode lignin content of cv. Harvest was over 23 % higher than that observed in cv. Kane (Fig. 7a). Similarly, the internode cellulose content of cv. Harvest was over 9 % higher than that of cv. Kane. With respect to hemicellulose, the internode of cv. Kane exhibited ~6 % higher level than that of cv. Harvest. Waterlogging caused significant reduction in the amounts of both lignin (36 %) and cellulose (20 %) contents in the internode tissues; however, no effect was evident on the content of hemicellulose (Fig. 7b). It also led to 60 % decrease in the flag leaf lignin content, while causing 5 and 30 % increases in the contents of cellulose and hemicellulose, respectively (Fig. 7c).

### Hormone analysis

**Fig. 8:**
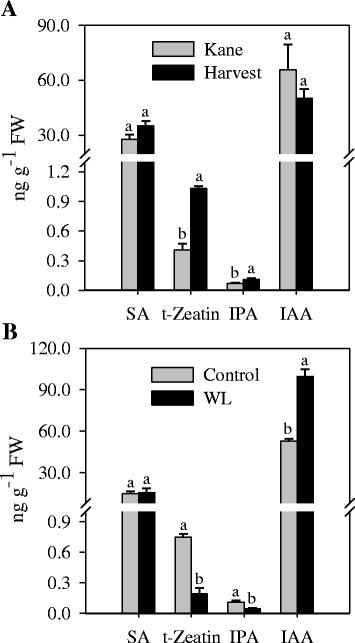
Profiles of salicylic acid (SA), cytokinin (t-zeatin and IPA), and auxin (IAA). Hormonal levels were determined in the internodes of the two wheat cultivars Kane and Harvest (**a**), and in the internode of cv. Harvest in response to waterlogging (WL) (**b**). Data are means of 2 to 3 independent biological replicates ± SE. Different *letters* within each hormone show statistically significant difference in level at *P* < 0.05. Plant materials to study differences in the levels of SA, t-zeatin, IPA and IAA between the two cultivars were grown in Super Mix (see Methods) while plant materials to study the effect of waterlogging on the levels of SA, t-zeatin, IPA and IAA were grown in soil mixture of clay and sand (2:1, v:v)

To examine the association between the levels of lignin and plant hormones that are implicated in the regulation of lignin biosynthesis, we measured the amounts of IAA, IPA and t-zeatin, and SA in the internode tissues. The levels of IPA, t-zeatin and SA in the internode of cv. Harvest were found to be higher than that observed in the internode of cv. Kane while the IAA content did not show any difference (Fig. 8a). Waterlogging, which decreased lignin content (Fig. 7c), led to a significant reduction in IPA and t-zeatin levels. In contrast, the level of IAA increased in response to waterlogging while no change in SA content was evident (Fig. 8b).

## Discussion

In order to gain insights into the molecular basis for the regulation lignin biosynthesis in wheat tissues, this study first examined the expression of lignin biosynthetic genes in different wheat tissues and then compared the expression patterns of selected genes in the internode tissue between two different wheat cultivars exhibiting different degree of resistance to lodging. The study also investigated the effect of waterlogging on the expression of lignin biosynthetic genes and the level of major lignocellulosic constituents. Furthermore, we assessed if lignin content is associated with the level of plant hormones implicatied in regulating lignin biosynthesis in other species.

Our gene expression data indicated high transcript abundance of *PAL6*, *C4H1*, *4CL1*, *HCT1*, *C3H1*, *CCoAOMT1*, *CCR2*, *CCR5*, *F5H2*, *COMT2* and *CAD2* genes in wheat tissues (Table 2; Additional file 6: Table S2), thus, it is more likely that these genes play important roles in the regulation of lignin biosynthesis in wheat. These results are in agreement with the expression patterns of lignin biosynthetic genes reported previously in both wheat and Arabidopsis [3, 18]. Within the stem, older internodes appeared to have higher expression for most of the candidate genes than the younger internode sections. Consistently, the expressions of selected lignin biosynthetic genes and lignin level have been shown to increase with internode age [3]. Although a specific member in each gene family exhibits predominance in expression across the different wheat tissues, two members of the *CCR* gene family showed tissue specificity; *CCR5* is predominantly expressed in the flag leaf while *CCR2* in the internode (Table 2). It has also been shown previously that the gene we designated as *CCR2* exhibits higher expression in the internode [21].

We further compared the expression of the candidate genes in two wheat cultivars exhibiting varying degrees of resistance to lodging, namely Harvest and Kane; cv. Harvest is designated agronomically as more resistant to lodging than cv. Kane. The higher internode lignin content of cv. Harvest than that of cv. Kane (Fig. 7a) suggests a close association between stem mechanical strength/lodging resistance and stem lignin content [12, 22]. The association of internode lignin content of cv. Harvest with the expression of *4CL1*, *CCR2*, *F5H2* and *COMT2* genes reflects the significance of these specific genes in regulating internode lignin content, which contributes to stem mechanical strength/lodging resistance in wheat. A close association between wheat stem lignin content and the expression of *CCR* and *COMT* genes and activities of the corresponding enzymes has also been reported previously [12, 21]. However, the difference in lodging resistance between the two cultivars may also be at least partly due to other factors that contribute to stem stiffness and thickness than internode lignin content, such as densities of cellulose and hemicellulose, structural properties of the secondary cell wall, and structural traits associated with cortical fiber tissues [52–54]. Consistently, the enhanced culm mechanical strength in a lignin deficient rice variety a has been shown to be associated with high densities of cellulose and hemicellulose, well developed secondary cell walls and thick layer of cortical fiber tissues [53].

Lignin biosynthesis in plants is affected by both biotic and abiotic stress factors [55]. Waterlogging is among the abiotic stress factor that triggers reduction of leaf photosynthetic capacity [56, 57] and accumulation of soluble carbohydrate in plant biomass [27]. Furthermore, it triggers increased demand for soluble sugars, particularly glucose [58, 59], which might lead to the channeling of more phosphoenol pyruvate to glycolysis rather than to the shikimate pathway that produces phenylalanine, an important lignin precursor [20]. These changes have important implications on the level and/or composition of structural carbohydrate polymers such as cellulose, hemicellulose and lignin [60, 61]. Consistently, the level of lignin in both internode and flag leaf tissues was substantially reduced in response to waterlogging (Fig. 7b, c). In the internode, waterlogging also caused a significant reduction in cellulose content; leading to a decrease in the total amount of structural carbohydrates. Since the synthesis of structural carbohydrate components such as lignin is an energy-consuming and irreversible process [62, 63], reduction in their level might be one of the energy/carbon saving strategies employed by plants under waterlogging conditions [24, 25]. In the flag leaf, the decrease in lignin content appears, however, to be compensated by significant increases in the levels of cellulose and hemicellulose (Fig. 7b).

Among the genes identified as highly expressed in wheat biomass, transcriptional repression of *PAL6*, *CCR2* and *F5H2* was apparent specifically in the internode of waterlogged wheat plants (Fig. 4b), suggesting the importance of these genes in modulating lignin formation in the internode under such stress conditions. Consistent with the lignin profile and expression of the *PAL6* gene, waterlogging caused reduction in the activity of internode derived PAL (Fig. 6a), an enzyme catalyzing the first committed step in the general phenylpropanoid pathway, and thereby controling lignin synthesis [15, 16]. In contrast, waterlogging mediated reduction of lignin content in both internode and leaf tissues is accompanied by upregulation of *PAL1*, *2*, *3* and *5* in the internode and *PAL6* in the leaf; upregulation of *PAL6* in the leaf is associated with increased activity of the PAL enzyme. Given that individual *PAL* genes respond differentially to abiotic stressors [64] and some *PAL* genes have been implicated in the synthesis of other phenylpropanoid-derived molecules such as tannins and anthocyanin [65, 66], which are known to function in diverse stress responses [67, 68], it is likely that the *PAL* genes upregulated in the tissues of waterlogged plants play a role for enhanced production of molecules that provide protection against waterlogging stress. Since the *PAL1*, *2*, *3* and *5* genes of waterlogged internodes are upregulated while the PAL activity is substantially reduced, we cannot rule out the possibility that these genes are subjected to post-transcriptional regulation. In support of this, Kelch repeat F-box (KFB) proteins mediated ubiquitination and degradation of the PAL isozymes have been shown to occur in Arabidopsis [69]. Furthermore, mapping of phosphopeptides in the PAL of *Zea mays* and *Medicago truncatula* has led to the speculation that PAL activity can be regulated post-transcriptionally by phosphorylation [70].

Waterlogging, which markedly reduced lignin content, also induced ≥2-fold upregulation of several genes downstream of *PAL* including *C4H1*, *HCT1* and *CCoAOMT1* in the internode and *C4H1*, *4CL1*, *HCT1*, *CCoAOMT1*, *F5H2*, *COMT2*, *CAD1* and *CAD3* genes in the leaf (Fig. 4); and the induction of *CAD1* and *CAD3* in the leaf is associated with increased activity CAD (Fig. 6b). It has been shown in previous studies that phenylpropanoid/monolignol pathway intermediates can serve as precursors not only for lignin but also for a range of secondary metabolites such as flavonoids, coumarins, stilbenes, hydroxycinnamic acid conjugates and lignans that play important roles in stress-related processes [64, 66]. Under stress conditions, when photosynthesis is sub-optimal, plants may re-direct the phenylpropanoid/monolignol pathway intermediates towards the production of these secondary metabolites [64]. Therefore, waterlogging-induced upregulation of the several lignin biosynthesis genes downstream of *PAL* in both internode and leaf tissues while lignin level is reduced might suggest the role of these genes for increased synthesis of anti-stress secondary metabolites at the expense of lignin to help the plants overcome the adverse effects of waterlogging. In support of this hypothesis, some isoforms of the enzymes or family members of the genes downstream of *PAL* have been implicated in the synthesis of phenylpropanoid-derived secondary metabolites [66, 71]. However, the possibility for post-transcriptional regulation of these downstream monolignol pathway genes can not be excluded as a recent study in poplar has shown that the activity of 5-hydroxyconiferaldehyde *O*-methyltransferase (AldOMT)/COMT can be regulated by phosphorylation [72], and the CAD and CCR of some species contain phosphopeptides that act as sites for phosphorylation mediated post-transcriptional regulation [70].

Previous studies have provided important insights into the role of different plant hormones in regulating the synthesis of lignin in plant tissues. For example, the levels of SA and lignin have been shown to have inverse relationship in plants where lignin content is reduced through down regulation of lignin biosynthetic genes, and SA was reported to mediate growth reduction in these plants [34]. However, genetic reduction of SA level restores growth but not the lignin content. The presence of similar amounts of SA between the internodes of cvs. Harvest and Kane (Fig. 8a) or between those derived from waterlogged and control plants (Fig. 8b) irrespective of differences in their lignin content might implicate that SA does not directly affect lignin formation. Close association between IAA and lignin levels has been reported previously in which IAA induces the expression of lignin biosynthetic genes and activity of the corresponding enzymes, and thereby lignification [30, 32]. However, internodes of the two cultivars with varying lignin content exhibit similar IAA level (Fig. 8a). Furthermore, internodes of waterlogged wheat plants, which exhibit reduced lignin content (Fig. 7b), contain higher amount of IAA than the internodes of control plants (Fig. 8b). These results might imply the significance of IAA signaling rather than IAA level in the control of lignin formation in wheat stem. Consistent with their role in enhancing lignin synthesis [30, 32], the levels of two bioactive cytokinins, IPA and t-zeatin, are directly associated with internode lignin content, suggesting that the level of liginin in wheat biomass can be optimized through manipulation of the level of cytokinin, IPA and t-zeatin. Since these plant hormones can also influence other plant developmental processes that potentially affect cell wall propreties and composition [73], further studies are needed to examine their specific physiological roles in determining lignin formation in wheat tissues. A recent report has also implicated other hormones such as absicic acid, brassinosteroids, jasmonates and ethylene in regulating cell wall properties and compoistion [73, 74]. Given that plant hormones interact either synergistically or antagonstically in regulating a wide range of plant developmental processes [75], it is important to elucidate molecular mechanisms underlying the role of hormonal interactions in the control of lignin synthesis in wheat tissues.

## Conclusions

This study showed that among the candidate lignin biosynthetic genes that are highly expressed in wheat tissues, the expressions of *4CL1*, *C3H1*, *CCR2*, *F5H2* and *COMT2* appear to be associated with internode lignin content, which contributes at least partly to lodging resistance. Reduction of the lignin content in the internode of waterlogged wheat plants appeared to be mediated by transcriptional repression of *PAL6*, *CCR2* and *F5H2* genes, implying the significance of these genes in modulating lignin level under such stress conditions. Furthermore, changes in internode lignin content are shown to be accompanied by changes in the level of cytokinin, IPA and t-zeatin, suggesting the role of cytokinin in the regulation of lignin deposition in wheat biomass. Given that mechanical strength/resistance to lodging, tolerance to biotic and abiotic stressors and feedstock quality of wheat straw are closely associated with lignin content, the findings of this study provides important insights into our understanding of the molecular mechanisms underlying lignin formation in wheat biomass. This is an important initial step towards the development of molecular tools that can facilitate the breeding of wheat cultivars with optimized lignin content, enhancing the feedstock quality of wheat straw without affecting lignin-associated agronomic traits.

### Availability of supporting data

The datasets supporting the conclusions of this article are included within the article and its additional files.

## Additional files

Additional file 1: Table S1. Primer sequences used for qPCR assays of specific lignin biosynthetic genes. Primer sequences were designed for the newly identified candidate lignin biosynthetic genes or redesigned in cases where previously reported primers [3] do not exhibit target gene specificity. (PDF 12 kb) Additional file 2: Figure S1. Phylogenetic relationships of wheat *PAL* and *C4H* genes with the homologs from other species. Phylogenetic trees of *PAL* (A) and *C4H* (B) were generated based on nucleic acid sequence similarity of the wheat genes with 28 *PAL* and 9 *C4H* genes, respectively, of other monocot and dicot species identified from the NCBI nucleotide database [39] using MEGA program [41], and the trees were inferred using Maximum Likelihood method based on the Tamura-nei model. The percentage of replicate trees in which the associated taxa clustered together in the bootstrap test of 500 replicates is shown next to the branches. ●, wheat candidate gene; ▲, genes from dicot species other than Arabidopsis; *, wheat sequence used for the analysis. (PDF 176 kb) Additional file 3: Figure S2. Phylogenetic relationships of wheat *4CL* and *HCT* genes with the homologs from other species. Phylogenetic trees of *4CL* (A) and *HCT* (B) were generated based on nucleic acid sequence similarity of wheat genes with 20 *4CL* and 9 *HCT* genes, respectively, of other monocot and dicot species identified from the NCBI nucleotide database [39] using MEGA program [41], and the trees were inferred using Maximum Likelihood method based on the Tamura-nei model. The percentage of replicate trees in which the associated taxa clustered together in the bootstrap test of 500 replicates is shown next to the branches. ●, wheat candidate gene; ▲, genes from dicot species other than Arabidopsis; *, wheat sequence used for the analysis. (PDF 175 kb) Additional file 4: Figure S3. Phylogenetic relationships of wheat *C3H* and *CCoAOMT* genes with the homologs from other species. Phylogenetic trees of *C3H* (A) and *CCoAOMT* (B) were generated based on nucleic acid sequence similarity of wheat genes with 15 *C3H* and 19 *CCoAOMT* genes, respectively, of other monocot and dicot species identified from the NCBI nucleotide database [39] using MEGA program [41], and the trees were inferred using Maximum Likelihood method based on the Tamura-nei model. The percentage of replicate trees in which the associated taxa clustered together in the bootstrap test of 500 replicates is shown next to the branches. ●, wheat candidate gene; ▲, genes from dicot species other than Arabidopsis; *, wheat sequence used for the analysis. (PDF 175 kb) Additional file 5: Figure S4. Phylogenetic relationships of wheat *F5H* and *CAD* genes with the homologs from other species. Phylogenetic trees *F5H* (A, B) and *CAD* (C) were generated based on nucleic acid sequence similarity of wheat genes with 14 *F5H* and 22 *CAD* genes, respectively, of other monocot and dicot species identified from the NCBI nucleotide database [39] using MEGA program [41], and the trees were inferred using Maximum Likelihood method based on the Tamura-nei model. The percentage of replicate trees in which the associated taxa clustered together in the bootstrap test of 500 replicates is shown next to the branches. Two separate phylogenetic trees were generated for wheat *F5H1* (A) and *F5H2* (B) genes as MEGA could not find common sites between *F5H1* and *F5H2*. ●, wheat candidate gene; ▲, genes from dicot species other than Arabidopsis; *, wheat sequence used for the analysis. (PDF 178 kb) Additional file 6: Table S2. Relative transcript levels of candidate lignin biosynthesis genes in different tissues of wheat. Transcript levels in different tissues of cv. Harvest were expressed relative to that of *4CL1* in flag leaf, which was arbitrarily set to value of 1. Data are means of 2 to 3 independent biological replicates ± SE. Means followed by different letters within each gene show statistically significant difference at *P* < 0.05. nd, not detected; FB, flag leaf blade; FS, flag leaf sheath; PE, peduncle; IN-1, the first internode; IN-2&3, the second and third internode; IN-4, the fourth internode (see the Methods section for numerical designation of internodes). (PDF 106 kb)

## Abbreviations

ADF: acid detergent fiber
CAD: cinnamyl alcohol dehydrogenase
CAD-CA: coniferyl aldehyde recognizing CAD
CAD-SA: sinapyl aldehyde recognizing CAD
IN: internode
NDF: neutral detergent fiber
G: guaiacyl unit of lignin
H: hydroxyphenyl unit of lignin
S: syringyl unit of lignin
