# Supplementary material for: Lignin biosynthesis in wheat (Triticum aestivum L.): its response to waterlogging and association with hormonal levels
Source: BMC Plant Biol. 2016 Jan 25;16:28. doi: 10.1186/s12870-016-0717-4 (PMC4727291; doi:10.1186/s12870-016-0717-4)
Supplement: Additional file 1: Table S1. — Primer sequences used for qPCR assays of specific lignin biosynthetic genes. Primer sequences were designed for the newly identified candidate lignin biosynthetic genes or redesigned in cases where previously reported primers [3] do not exhibit target gene specificity. (PDF 12 kb) [file 12870_2016_717_MOESM1_ESM.pdf]

Table S1. Primer sequences used for qPCR assays of specific lignin biosynthetic genes[a]

Genes	Type	Primer (5' to 3')
PAL1	Forward	GCTTGGCAAAGACGGATGTC
	Reverse	AGCGCCAACTACCCATTGAT
PAL6	Forward	CTCAAGCTCATGTCCTCCACA
	Reverse	TCAGCACCTTCTTCGACACC
C3H2	Forward	AAGCCACTGGTTGTCAGGAG
	Reverse	TGAACTCCTGCCCTTCTTCG
C3H3	Forward	AAGATCATGGAGGAGCACGC
	Reverse	CGGTGATCATGTCCCAGAGG
CCR2	Forward	GAAACAGCGCGGTTCACGTT
	Reverse	AGGAATGCTGGCAACAAACCC
CCR6	Forward	TGACTGACGATCCAGAGCAG
	Reverse	GCAGAATTCAAGGTCGCTCC
COMT1	Forward	GGTTCGCCGCCATGAAGACTA
	Reverse	CAGGTGGATGCATCAGAGAGGTA
COMT2	Forward	GTCCGCCGATCTGAGAATCTTC
	Reverse	CGACGACACAACCAGTAGAG
COMT3	Forward	GAGTATCATGGCACGGACCC
	Reverse	ACGCCATTGAAGCCCTTGTA
CAD1	Forward	TTACCTACCAGCTCCAGCCA
	Reverse	TCTGGAATTGCACGCACCAA

[a]Primer sequences were designed for the newly identified candidate lignin biosynthetic genes or redesigned in cases where previously reported primers [3] do not exhibit target gene specificity.
