# Supplementary material for: Lignin biosynthesis in wheat (Triticum aestivum L.): its response to waterlogging and association with hormonal levels
Source: BMC Plant Biol. 2016 Jan 25;16:28. doi: 10.1186/s12870-016-0717-4 (PMC4727291; doi:10.1186/s12870-016-0717-4)

Figure S2. Phylogenetic relationships of wheat *4CL* and *HCT* genes with the homologs from other species. Phylogenetic trees of *4CL* (A) and *HCT* (B) were generated based on nucleic acid sequence similarity of wheat genes with 20 *4CL* and 9 *HCT* genes, respectively, of other monocot and dicot species collected from the NCBI nucleotide database [39] using MEGA program [41], and the trees were inferred using Maximum Likelihood method based on the Tamura-nei model. The percentage of replicate trees in which the associated taxa clustered together in the bootstrap test of 500 replicates is shown next to the branches. ●, wheat candidate gene; ▲, genes from dicot species other than Arabidopsis; *, wheat sequence used for the analysis.

A

B

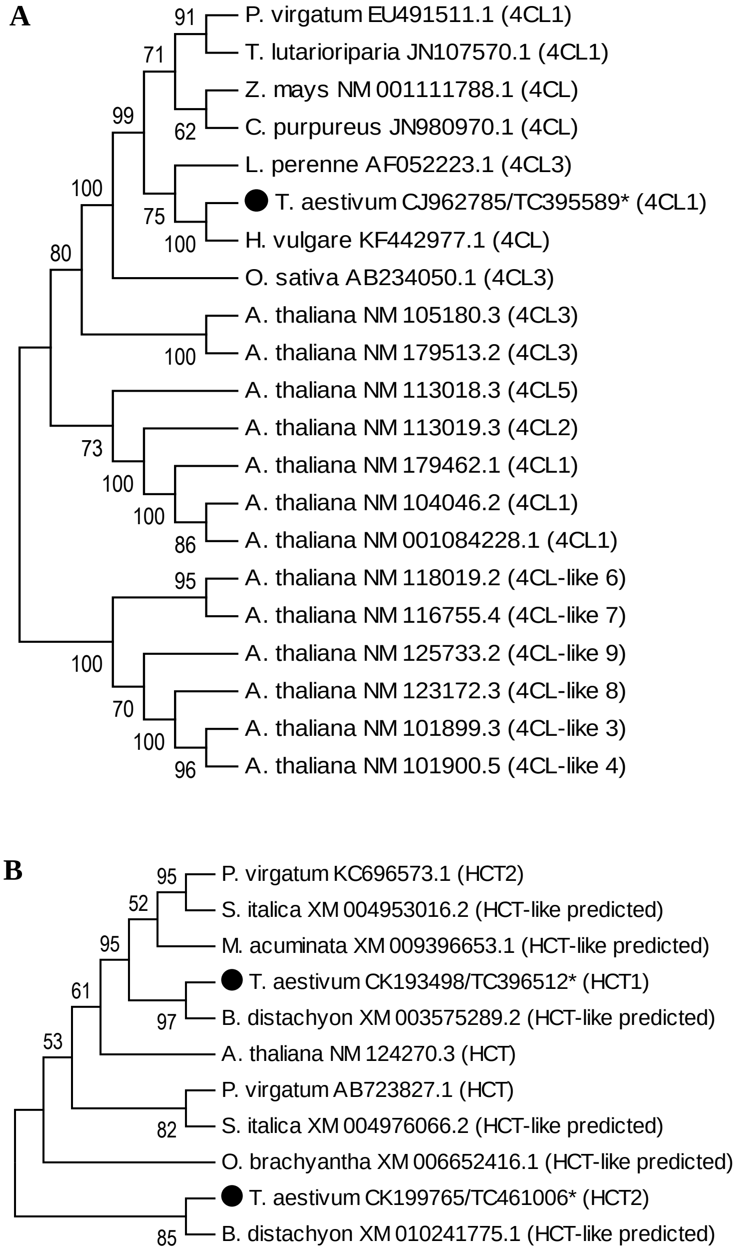

Supplement: Additional file 3: Figure S2. — Phylogenetic relationships of wheat 4CL and HCT genes with the homologs from other species. Phylogenetic trees of 4CL (A) and HCT (B) were generated based on nucleic acid sequence similarity of wheat genes with 20 4CL and 9 HCT genes, respectively, of other monocot and dicot species identified from the NCBI nucleotide database [39] using MEGA program [41], and the trees were inferred using Maximum Likelihood method based on the Tamura-nei model. The percentage of replicate trees in which the associated taxa clustered together in the bootstrap test of 500 replicates is shown next to the branches. ●, wheat candidate gene; ▲, genes from dicot species other than Arabidopsis; *, wheat sequence used for the analysis. (PDF 175 kb) [file 12870_2016_717_MOESM3_ESM.pdf]
