# Supplementary material for: Lignin biosynthesis in wheat (Triticum aestivum L.): its response to waterlogging and association with hormonal levels
Source: BMC Plant Biol. 2016 Jan 25;16:28. doi: 10.1186/s12870-016-0717-4 (PMC4727291; doi:10.1186/s12870-016-0717-4)

Figure S4. Phylogenetic relationships of wheat *F5H* and *CAD* genes with the homologs from other species. Phylogenetic trees of *F5H* (A, B) and *CAD* (B) were generated based on nucleic acid sequence similarity of wheat genes with 7 *F5H* and 22 *CAD* genes, respectively, of other monocot and dicot species collected from the NCBI nucleotide database [39] using MEGA program [41], and the trees were inferred using Maximum Likelihood method based on the Tamura-nei model. The percentage of replicate trees in which the associated taxa clustered together in the bootstrap test of 500 replicates is shown next to the branches. ●, wheat candidate gene; ▲, genes from dicot species other than Arabidopsis; *, wheat sequence used for the analysis. Two separate phylogenetic trees were generated for wheat *F5H1* (A) and *F5H2* (B) genes as MEGA could not find common sites between *F5H1* and *F5H2*.

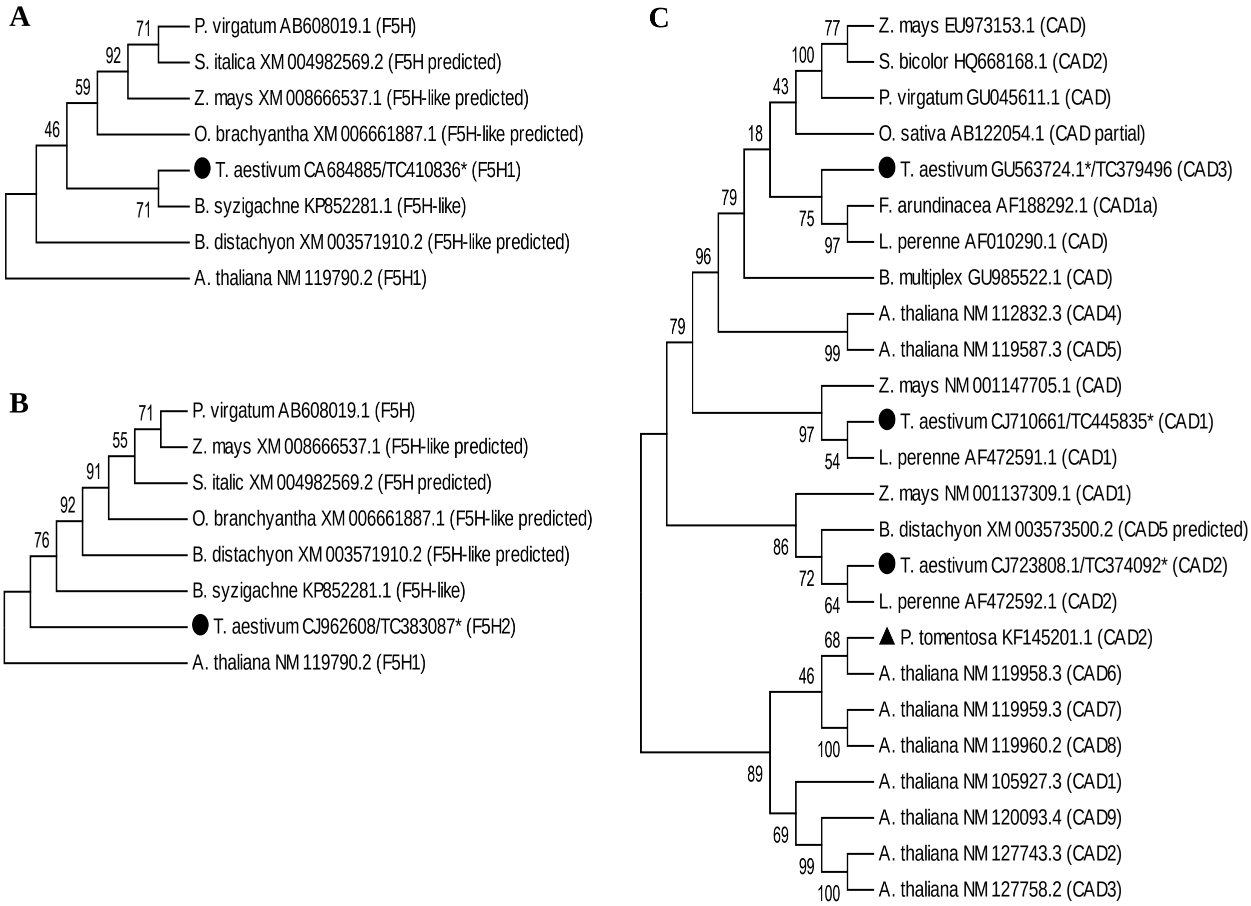

Supplement: Additional file 5: Figure S4. — Phylogenetic relationships of wheat F5H and CAD genes with the homologs from other species. Phylogenetic trees F5H (A, B) and CAD (C) were generated based on nucleic acid sequence similarity of wheat genes with 14 F5H and 22 CAD genes, respectively, of other monocot and dicot species identified from the NCBI nucleotide database [39] using MEGA program [41], and the trees were inferred using Maximum Likelihood method based on the Tamura-nei model. The percentage of replicate trees in which the associated taxa clustered together in the bootstrap test of 500 replicates is shown next to the branches. Two separate phylogenetic trees were generated for wheat F5H1 (A) and F5H2 (B) genes as MEGA could not find common sites between F5H1 and F5H2. ●, wheat candidate gene; ▲, genes from dicot species other than Arabidopsis; *, wheat sequence used for the analysis. (PDF 178 kb) [file 12870_2016_717_MOESM5_ESM.pdf]
