# Supplementary material for: Lignin biosynthesis in wheat (Triticum aestivum L.): its response to waterlogging and association with hormonal levels
Source: BMC Plant Biol. 2016 Jan 25;16:28. doi: 10.1186/s12870-016-0717-4 (PMC4727291; doi:10.1186/s12870-016-0717-4)
Supplement: Additional file 6: Table S2. — Relative transcript levels of candidate lignin biosynthesis genes in different tissues of wheat. Transcript levels in different tissues of cv. Harvest were expressed relative to that of 4CL1 in flag leaf, which was arbitrarily set to value of 1. Data are means of 2 to 3 independent biological replicates ± SE. Means followed by different letters within each gene show statistically significant difference at P < 0.05. nd, not detected; FB, flag leaf blade; FS, flag leaf sheath; PE, peduncle; IN-1, the first internode; IN-2&3, the second and third internode; IN-4, the fourth internode (see the Methods section for numerical designation of internodes). (PDF 106 kb) [file 12870_2016_717_MOESM6_ESM.pdf]

Table S2. Relative transcript levels of candidate lignin biosynthesis genes in different tissues of wheat[w]

Gene	FB	FS	PE	IN-1	IN-2&3	IN-4
PAL1	0.34 ±0.08[x]b[y]	0.02 ±0.00c	0.01 ±0.00d	0.25 ±0.06b	2.18 ±0.25a	2.40 ±0.29a
PAL2	0.26 ±0.05cd	0.16 ±0.02d	0.36 ±0.10c	0.75 ±0.13b	2.76 ±0.22a	3.22 ±0.23a
PAL3	0.26 ±0.03c	0.12 ±0.01d	0.16 ±0.03d	0.32 ±0.05c	1.48 ±0.16b	2.35 ±0.30a
PAL4	0.54 ±0.03d	1.58 ±0.16c	7.92 ±0.60a	6.51 ±0.70b	6.95 ±1.07ab	7.28 ±0.76a
PAL5	0.32 ±0.03e	0.28 ±0.01e	0.49 ±0.08d	0.89 ±0.04c	3.80 ±0.44b	5.71 ±0.65a
PAL6	0.83 ±0.07d	9.05 ±0.80c	89.25 ±19.53a	54.50 ±6.76b	54.57 ±11.98b	33.66 ±6.98b
C4H1	0.38 ±0.03d	0.91 ±0.04c	2.68 ±0.40b	3.67 ±0.61b	9.57 ±1.20a	11.22 ±3.71a
4CL1	1.00 ±0.09d	2.04 ±0.08c	7.61 ±1.10b	7.13 ±0.57b	12.51 ±2.02a	16.84 ±2.83a
HCT1	0.58 ±0.03d	0.70 ±0.06d	2.53 ±0.54b	1.54 ±0.20c	3.09 ±0.40b	5.42 ±0.60a
HCT2	0.19 ±0.02d	0.31 ±0.02d	2.77 ±0.72a	0.85 ±0.09c	1.35 ±0.10b	2.18 ±0.40ab
C3H1	0.12 ±0.03e	0.21 ±0.03de	1.24 ±0.06a	0.87 ±0.12b	0.55 ±0.13c	0.46 ±0.03cd
C3H2	nd[z]	nd	nd	0.01 ±0.00	nd	nd
C3H3	0.06 ±0.01d	0.35 ±0.16b	0.92 ±0.26a	0.31 ±0.01b	0.24 ±0.09bc	0.15 ±0.02c
CCoAOMT1	1.52 ±0.17e	3.32 ±0.58d	32.73 ±5.28a	11.98 ±2.51c	19.56 ±3.50bc	21.89 ±4.77b
CCoAOMT2	0.02 ±0.00c	0.05 ±0.00c	0.03 ±0.00a	0.02 ±0.00ab	0.02 ±0.00c	0.02 ±0.00bc
CCoAOMT3	0.72 ±0.04d	0.94 ±0.07cd	6.35 ±1.81a	1.24 ±0.13c	3.15 ±0.56b	5.04 ±1.47ab
CCR1	11.11 ±0.21a	6.64 ±0.36b	0.29 ±0.03f	1.59 ±0.31c	0.78 ±0.02d	0.45 ±0.07e
CCR2	0.17 ±0.02e	1.56 ±0.07d	3.77 ±0.78c	5.93 ±0.29b	8.44 ±0.85a	7.82 ±1.09a
CCR3	0.03 ±0.00cd	0.01 ±0.00e	0.02 ±0.00de	0.06 ±0.00bc	0.22 ±0.08ab	0.56 ±0.01a
CCR4	0.02 ±0.01b	0.01 ±0.01b	0.01 ±0.00b	0.03 ±0.02b	0.03 ±0.01b	0.12 ±0.00a
CCR5	13.49 ±1.10a	9.33 ±0.21b	0.93 ±0.09d	2.76 ±0.66cd	2.85 ±0.36cd	3.54 ±0.63c
CCR6	nd	0.01 ±0.00d	0.03 ±0.00c	0.08 ±0.02ab	0.06 ±0.00b	0.11 ±0.01a
F5H1	0.44 ±0.13c	0.37 ±0.07cd	4.56 ±0.80a	0.80 ±0.09b	0.20 ±0.03d	0.55 ±0.09bc
F5H2	0.84 ±0.04e	1.39 ±0.04d	9.20 ±1.67a	6.09 ±0.97ab	3.38 ±1.05c	4.23 ±0.84bc
COMT1	0.01 ±0.00f	1.28 ±0.27c	6.06 ±1.16a	2.82 ±0.32b	0.34 ±0.09d	0.16 ±0.02e
COMT2	15.51 ±1.13b	36.04 ±4.20a	16.09 ±2.86b	31.56 ±4.40a	14.18 ±1.64b	12.63 ±0.67b
COMT3	0.14 ±0.02c	0.21 ±0.02c	1.97±0.24a	0.90 ±0.23b	0.87 ±0.10b	0.64 ±0.21b
CAD1	1.40 ±0.01ab	1.00 ±0.03bc	0.38 ±0.09c	0.61 ±0.12c	1.81 ±0.23a	1.61 ±0.34a
CAD2	1.32 ±0.07b	2.05 ±0.08a	0.16 ±0.00c	0.95 ±0.22b	2.02 ±0.17a	2.55 ±0.29a
CAD3	0.84 ±0.10abc	1.15 ±0.10ab	1.36 ±0.37a	1.10 ±0.25ab	0.70 ±0.04bc	0.42 ±0.05c

[w]Transcript levels in different tissues of cv. Harvest were expressed relative to that of *4CL1* in flag leaf, which was arbitrarily set to value of 1.

[x]Data are means of 2 to 3 independent biological replicates ± SE.

[y]Means followed by different letters within each gene show statistically significant difference at $P<0.05$.

[z]nd, not detected

FB, flag leaf blade; FS, flag leaf sheath; PE, peduncle; IN-1, the first internode; IN-2&3, the second and third internode; IN-4, the fourth internode (see the materials and methods section for numerical designation of internodes).
